# Association between physical activity and all-cause mortality in patients with depression: a prospective cohort study based on NHANES data

**DOI:** 10.3389/fpubh.2025.1518255

**Published:** 2025-04-16

**Authors:** Jiaqiang Xiao, Xiaosheng Dong, Meng Ding, Qingqing Yang, Tao Kong

**Affiliations:** ^1^College of Physical Education, Shandong Normal University, Jinan, China; ^2^Department of Social Medicine and Health Management, School of Public Health, Cheeloo College of Medicine, Shandong University, Shandong, China; ^3^NHC Key Lab of Health Economics and Policy Research (Shandong University), Shandong, China; ^4^Center for Health Management and Policy Research, Shandong University (Shandong Provincial Key New Think Tank), Shandong, China; ^5^Institute of Health and Elderly Care, Shandong University, Shandong, China

**Keywords:** physical activity, depression, mortality, cohort study, NHANES

## Abstract

**Objective:**

This prospective cohort study aimed to investigate the association between physical activity (PA) and all-cause mortality in patients with depression.

**Methods:**

Data from 2,841 subjects were derived from the 2005–2018 U.S. National Health and Nutrition Examination Survey (NHANES), which included 13 years of follow-up. Depression was assessed using the Patient Health Questionnaire-9 (PHQ-9). The relationships between different amounts and types of physical activity (PA, such as work, transport, or leisure) and all-cause mortality were analyzed using multivariate Cox proportional hazard regression models and restricted cubic splines.

**Results:**

After adjusting for all covariates, the depressed patients who engaged in sufficient PA (≥600 metabolic equivalent (600 MET)-min/week) showed a 40% (hazard ratio [HR] = 0.60, 95% confidence interval [CI]: 0.47–0.76) lower mortality risk compared to their physically inactive counterparts. The mortality rate from recreational PA continues to decline with increasing proportions. Subgroup analyses further revealed sustained benefits in vulnerable populations: stroke patients maintaining sufficient PA achieved a HR of 0.40 (95% CI: 0.18–0.88) for all-cause mortality, while those with cardiac conditions showed an even more pronounced HR of 0.35 (95% CI: 0.16–0.77).

**Conclusion:**

PA has a positive effect on reducing the risk of death in patients with depression, and there are differences in the effectiveness of different volumes and purposes (for work, leisure, or transport) of PA in relation to reducing the risk of death. These findings emphasize the critical role of PA in mitigating mortality risk among individuals with depression, promoting personalized exercise plans that consider differences in activity volume and purposes.

## Highlights

- Depression is associated with higher all-cause mortality rates.
- Physical activity has been shown to be beneficial to physical and mental health.
- Physical activity (PA) reduces all-cause mortality in depressed patients.
- Different physical activities impact the effect of reducing all-cause mortality.
- As more and more people have a strong interest and expectation in the role of physical activity as a substitute for drugs, it may become a beneficial measure to reduce the risk of death in patients with depression with” the lowest cost and good effect.”

## 1 Introduction

Depression is one of the world’s most serious mental health problems (1). It has become a major disease that has the same adverse consequences on health and society as diabetes and hypertension. Depression not only causes psychological deterioration in daily life activities (2) but also negatively affects mood, cognition, somatic symptoms, feelings, thinking, and behaviors (3). It is also associated with physical changes such as weight loss, dullness of the eyes, loss of appetite, and marked fatigue (4). Moreover, according to World Health Organization (WHO) statistics, there are approximately 322 million patients with depression worldwide, and the incidence of depression is constantly increasing. A meta-analysis demonstrated a strong correlation between depression and an increased risk of death during the follow-up period (5). Depression has been shown to increase mortality among patients in different countries (6–8). In addition, depression leads to high rates of associated disability, making it one of the leading causes of the global burden of disability (4). In the US, depression may cause a 52% (5) increased risk of death. Fabiano et al., in their two recent meta-analyses, looked at how exercise affects suicide attempts or the risk of death; in their conclusion, they showed that exercise reduces people’s suicide attempts (9). Depression is also a prevalent risk factor for cardiovascular disease (10) and is independently associated with increased cardiovascular disease and mortality (11, 12). Depression combined with hypertension creates a toxic combination, leading to an increased risk of all-cause mortality (13). In addition, depression causes an annual economic burden of approximately $118 billion (14) and a considerable financial burden on health care (15). The global burden of disease is expected to be the leading cause of depression (16). Therefore, the prevention and treatment of depression remain public health priorities.

For people with mild to moderate or severe depression, the efficacy of drug therapy may be limited (17). The success rates of antidepressant medications and psychotherapy are approximately 72 and 50% (18, 19), respectively; only 10–25% of patients are affected to receive treatment for depressive symptoms, as medication can be costly and of limited efficacy for the majority of people with depression, with more than 75% of people in low- and middle-income countries not receiving treatment (20). Moreover, only approximately 30% of those treated respond to initial treatment. Given the high disease burden and societal costs, research studies must focus on alternative therapies and improving treatment outcomes (21). Physical activity (PA) is capable of producing multiple benefits, including psychological well-being. To achieve practical health benefits, the WHO recommends that all adults perform 150–300 min of moderate-intensity physical activity, 75–150 min of vigorous-intensity physical activity, or some equivalent combination of moderate-intensity and vigorous-intensity aerobic physical activity per week (22). As an effective treatment strategy for depression (23), PA has been shown to reduce symptoms of depression and can be a valuable addition to medications and psychotherapy (24). A meta-analysis found that physical activity reduces the risk of depression, offering significant benefits even at levels below the recommended amount; however, additional benefits diminish at higher activity volumes (25). PA interventions are virtually free of side effects and high costs associated with antidepressant medications and psychotherapy (26). PA combats depression through dual mechanisms: biologically by improving neuroplasticity, reducing inflammation and oxidative stress, and regulating the hypothalamic–pituitary–adrenal (HPA) axis, and psychologically by boosting self-esteem, social support, and self-efficacy (27). Additionally, PA reduces mortality risk by enhancing cardiorespiratory fitness (VO₂ max), preventing chronic diseases (e.g., type-2 diabetes [T2D] and cardiovascular diseases [CVD]), and activating survival pathways (e.g., AMPK/PGC-1α-mediated mitochondrial biogenesis, BDNF-dependent neuroprotection) (28). As an increasing number of people have a strong interest and expectation in the role of PA as a substitute for drugs (29), it may become a beneficial measure to reduce the risk of death in patients with depression with “the lowest cost and good effect.”

However, the majority of the of the published articles have focused on examining the effects of PA in terms of the prevalence of depression. Although PA has been shown to reduce mortality in different populations (30–33), there are still few studies on the association between PA and the risk of death in patients with depression. Therefore, this study utilizes on the NHANES database, which provides a nationally representative sample, to analyze the relationship between PA and the risk of death in patients with depression through a prospective cohort study. We hypothesize that achieving the recommended levels of PA can reduce mortality in depressed patients.

## 2 Method

We used data from studies based on the publicly available U.S. National Health and Nutrition Examination Survey (NHANES: 2005–2018) population. National Health and Nutrition Examination Survey (NHANES), and their survival status was correlated with the U.S. National Death Index (NDI) database through the subject’s common serial number (SEQN). The analytic sample excluded individuals aged below 20 years who were not depressed and who did not have physical activity response data. All studies were approved by the institutional review boards of their coordinating agencies and adhered to the principles outlined in the Declaration of Helsinki.

### 2.1 Study population

The data for this study were obtained from the NHANES database in the United States, which is a national cross-sectional household interview survey conducted by the Centers for Disease Control and Prevention and the National Center for Health Statistics through the U.S. Census Bureau every 2 years beginning in 1999. The data were collected via Multiethnic Cohort (MEC) experimental vehicle measurements and interview questionnaires. The subjects were selected using a stratified, multistage probabilistic design and were representative of the US civilians living in units, regions, and communities. The basic health information, physical indices, and physiological and biochemical indices were collected from the subjects. A detailed description of the design and methodology is available on the NHANES official webpage. All participants provided written informed consent, and the protocol was approved by the Ethics Review Board of the National Center for Health Statistics (Protocols #98-12, #2005-06, and #2011-17).

**Figure 1:**
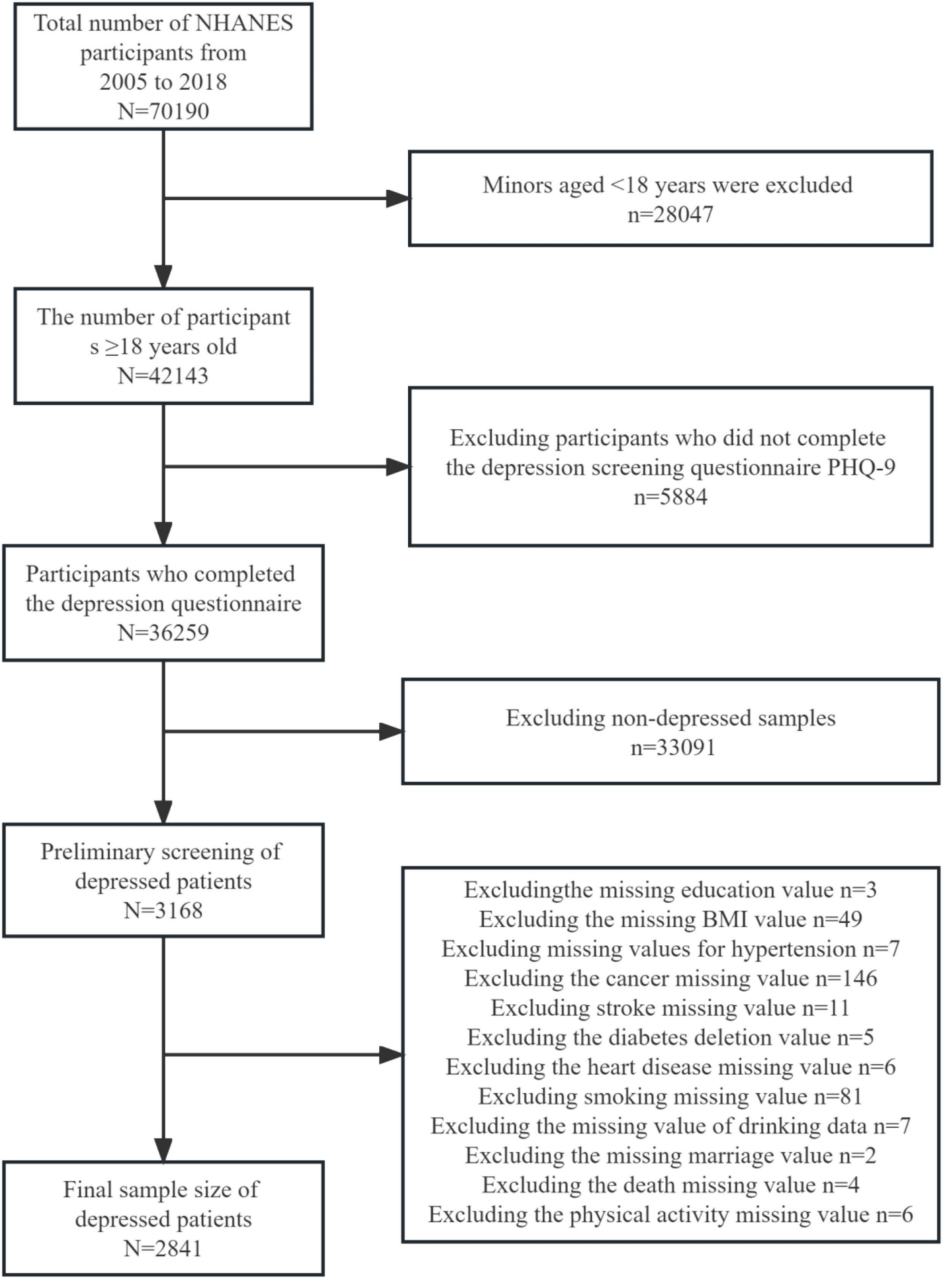
Flow chart of the screening of eligible participants.

This study included a representative sample of US population data collected between 2005 and 2018 linked to the National Death Index (NDI) as of 31 December 2019. A total of 70,190 subjects were surveyed during this period. The measurements were made via scores from the Depression Screening Health Questionnaire (PHQ-9), a 9-item depression screening tool, with depression clinically defined as a score of 10 or higher (34). In this study, those with depression scores above or equal to 10 in the data were determined to be depressed based on the final score, which had high sensitivity and specificity for the diagnosis of depression (34, 35). Exclusion criteria: (1) Age of the patients below 18 years; (2) Missing data on depression; (3) No diagnosed depression; (4) Missing data on physical activity; and (5) Missing data on covariates. Ultimately, 2,841 US adults with depression who completed the entire questionnaire were included in the statistical analyses. The subjects with missing data were those who were missing, were unsure, did not know the answer, or refused to answer the survey (Figure 1).

### 2.2 Measurement indicators

#### 2.2.1 Assessment of PA

Using the Global PA Questionnaire (GPAQ) developed by the World Health Organization (WHO) (36). GPAQ has been shown to be a reliable instrument for measuring the level of physical activity (36). PA was assessed through interviews. The questionnaire assessed self-reported routine PA for three different purposes (work activities, leisure activities, and daily transportation time) involving 10 min sustained activities in a typical week. The questionnaire also inquired about the duration of each activity and the frequency of occurrence per day, week, or month; it also considered the intensity at which the PA was performed (moderate or vigorous) and provided the corresponding MET value (37). The total metabolic equivalents per minute per week (MET-min/week) were calculated according to the physical activity volume (PAV) protocol. It is generally divided into two categories: insufficient PA (PAV < 600 MET-min/week) and sufficient PA (PAV ≥ 600 MET-min/week). Second, PAV ≥ 600 can be subdivided into six subcategories (600–1,199, 1,200–1,799, 1,800–2,999, 3,000–5,999, 6,000–8,999, and ≥ 9,000). The total PA is the sum of three different types of PAVs for various purposes. The PAV for each category of PA can be expressed in metabolic equivalents (METs) and is calculated by the formula: PAV = Provided MET × Weekly frequency × Time for each physical activity. In the restricted cubic spline (RCS) analysis presented in this article, the percentages of PAs divided into three categories for different purposes were compared and calculated as follows: PAVs for work/total PAVs, PAVs for leisure/total PAVs, and PAVs for transport/total PAVs.

#### 2.2.2 Assessment of the outcomes

Linking of eligible NHANES subjects from 2005 to 2018 to the NDI records updated as of 31 December 2019 (NDI official website). Further details of the matching method are available from the National Center for Health Statistics (NCHS Data Linkage > > Mortality Data > > Restricted-Use Data). To determine the survival status of NHANES subjects, we used RStudio software to create probabilistic records based on the unique identifier, SEQN, as well as the officially provided coding file, which was linked to the NDI records. The accuracy of all-cause and cause-specific mortality information in the NDI records has been validated in previous studies. The follow-up time was defined as the time from NHANES participation to the date of the decedent’s death or survivor review (31 December 2019). In this study, causes of death were coded using the *International Classification of Diseases, 10th Revision (ICD-10)*, in addition to all-cause mortality, which served as the primary outcome for mortality and was categorized as survivors or deceased. The ICD-10 deciphered codes for the official NDI download of the R file are shown in Box 1.

BOX 1 **ICD-10**

1 = Diseases of the heart (I00-I09, I11, I13, I20-I51)
2 = Malignant neoplasms (C00-C97)
3 = Chronic lower respiratory diseases (J40-J47)
4 = Accidents (unintentional injuries) (V01-X59, Y85-Y86)
5 = Cerebrovascular diseases (I60-I69)
6 = Alzheimer’s disease (G30)
7 = Diabetes mellitus (E10-E14)
8 = Influenza and pneumonia (J09-J18)
9 = Nephritis, nephrotic syndrome and nephrosis (N00-N07, N17-N19, N25-N27)
10 = All other causes (residual)
<NA> = Ineligible, under age 18, assumed alive, or no cause of death data available

ICD-10 = International Classification of Diseases, 10th Revision.

#### 2.2.3 Covariates

The sociological factors considered in this study were derived from the demographics of the sample data in terms of age (<65 or ≥ 65 years), sex (male or female), race (Mexican–American, other Hispanic, non-Hispanic White, non-Hispanic Black, and other race), marital status (live together, never married, and separated), educational attainment (high school, above high), body mass index (BMI) categorized as usual, overweight, and obese (<25 kg/m^2^, 25–30 kg/m^2^, and ≥ 30.0 kg/m^2^, respectively), smoking status (never, former, and current), drinking status (never, former, and current), medical diagnosis of chronic disease-related hypertension (yes/no), diabetes mellitus (yes or no), cancer (yes or no), heart disease (yes or no), and stroke (yes or no).

### 2.3 Statistical analyses

For this study, the baseline characteristics of the subjects are expressed as numbers (*N*) and percentages (%) through categorical variables. In addition, differences between the baseline characteristics of the subjects in terms of different variables between the two PA subgroups (PAV < 600 MET-min/week and PAV ≥ 600 MET-min/week) were tested via *t* tests and chi-square tests, and Cox proportional risk regression models were used to test the relationships between PA levels and the risk of all-cause mortality (HR and 95% CI). To analyze the proportion of PAs with different purposes, the data were collated using R software, and the non-linear relationship between PAs with different independent variables and mortality was initially confirmed through the code (non-linear *p-*value below 0.001). The RCS curves based on the Cox proportional risk model were subsequently generated on a continuous scale. The correlation between PAs and all-cause mortality was assessed. Three models for PA and mortality were developed to explore the quantitative effects of different potential confounding factors of covariates; adjusts for age and sex in Model 1, Model 2 adjusts for education level and body mass index (BMI), smoking status, and drinking status in Model 1, and Model 3 adjusts for the chronic disease covariates addressed in this article again based on Model 2. In addition, subgroup analyses were performed to test the associations with all-cause mortality risk through the interaction of potential covariates (age, sex, education level, race, marital status, BMI, smoking status, drinking status, and chronic disease status) with PA subgroups. In the subgroup analysis, the participants were grouped according to the same covariates as those in Section 2.2.3. A two-sided *p*-value below 0.05 was considered statistically significant. The above data were statistically analyzed using Statistical Package for the Social Sciences (SPSS) software (version 26.0, IBM Corp, Armonk, NY, United States) and RStudio software (version 4.2.3, R Foundation, for Statistical Computing, Vienna, Austria).

## 3 Results

### 3.1 Description of the study population

Among the 2,841 eligible adult patients with depression in the United States, 63.60% were female, and 17.63% were aged 65 years or older. 54.84% (*N* = 1,558) had insufficient PA (<600 MET-min/week), and 45.16% (*N* = 1,283) of the depressed patients had sufficient PA. Table 1 shows the baseline characteristics of the subjects. The percentage of female subjects was greater than that of male subjects in both PA subgroups: Sufficient PAs: 758 (59.08%) and 525 (40.92%); Insufficient PAs: 1,049 (67.33%) and 509 (32.67%). Subjects who had sufficient PA had a lower prevalence of hypertension (39.75% vs. 54.04%), cancer (9.04% vs. 12.90%), stroke (5.22% vs. 9.95%), heart disease (4.29% vs. 10.14%), and diabetes mellitus (13.72% vs. 25.48%) than those in the lack of PA group did, and the unmarried status of this subject group (26.66% vs. 15.60%), higher education level (45.13% vs. 36.20%) and normalized BMI (27.90% vs. 24.33%) were more prevalent.

**Table 1:** Baseline characteristics.

Characteristics	Overall sample (*N* = 2,841)		
	Insufficient PA (*N*/%)	Sufficient PA (*N*/%)	*p*-value
All patients	1,558 (54.84%)	1,283 (45.16%)	
Gender			<0.001
Men	509 (32.67%)	525 (40.92%)	
Women	1,049 (67.33%)	758 (59.08%)	
Age			<0.001
<65	1,208 (77.54%)	1,132 (88.23%)	
≥65	350 (22.46%)	151 (11.77%)	
Hypertension			<0.001
Yes	842 (54.04%)	510 (39.75%)	
No	716 (45.96%)	773 (60.25%)	
Cancer			0.001
Yes	201 (12.90%)	116 (9.04%)	
No	1,357 (87.10%)	1,167 (90.96%)	
Stroke			<0.001
Yes	155 (9.95%)	67 (5.22%)	
No	1,403 (90.05%)	1,216 (94.78%)	
Heart disease			<0.001
Yes	158 (10.14%)	55 (4.29%)	
No	1,400 (89.86%)	1,228 (95.71%)	
Diabetes			<0.001
Yes	397 (25.48%)	176 (13.72%)	
No	1,161 (74.52%)	1,107 (86.28%)	
Education			<0.001
Under high	618 (39.67%)	397 (30.94%)	
High school	376 (24.13%)	307 (23.93%)	
Above high	564 (36.20%)	579 (45.13%)	
Marital status			<0.001
Live together	718 (46.08%)	578 (45.05%)	
Never married	243 (15.60%)	342 (26.66%)	
Separated	597 (38.32%)	363 (28.29%)	
Smoking status			0.270
Never	666 (42.75%)	510 (39.75%)	
Former	337 (21.63%)	294 (22.92%)	
Current	555 (35.62%)	479 (37.33%)	
Drinking status			0.037
Never	223 (14.31%)	153 (11.93%)	
Former	347 (22.27%)	259 (20.19%)	
Current	988 (63.41%)	871 (67.89%)	
BMI			<0.001
<25	331 (21.24%)	354 (27.59%)	
≥25and < 30	379 (24.33%)	358 (27.90%)	
≥30	848 (54.43%)	571 (44.51%)	
Race			0.001
Mexican American	249 (15.98%)	187 (14.58%)	
Other Hispanic	201 (12.90%)	173 (13.48%)	
Non-Hispanic White	657 (42.17%)	530 (41.31%)	
Non-Hispanic Black	366 (23.49%)	272 (21.20%)	
Other race	85 (5.46%)	121 (9.43%)	

### 3.2 Association of PA levels with all-cause mortality in depressed patients

#### 3.2.1 Association of sufficient PA with all-cause mortality in depressed patients

Table 2 shows the correlations between sufficient physical activities and the risk of all-cause mortality. In the fully adjusted model (Model 3), patients with depression who engaged in sufficient physical activity (≥600 MET-min/week) exhibited a 40% lower risk of all-cause mortality compared to those with insufficient physical activity with HR = 0.60 (95% CI: 0.47–0.76).

**Table 2:** Association of different PA groups with all-cause mortality in depressed patients.

Variables	Model 1	Model 2	Model 3
Insufficient PA	Reference	Reference	Reference
Sufficient PA	0.52 (0.41–0.66)	0.55 (0.43–0.70)	0.60 (0.47–0.76)

Hazard ratios (95% confidence intervals).

Model 1: Adjusted for gender + Age.

Model 2: Adjusted for Model 1 + Education, BMI, smoking status, and drinking status.

Model 3: Adjusted for Model 2 + Chronic illness (hypertension, cancer, stroke, heart disease, and diabetes).

#### 3.2.2 Association of different PAVs with all-cause mortality in depressed patients

**Figure 2:**
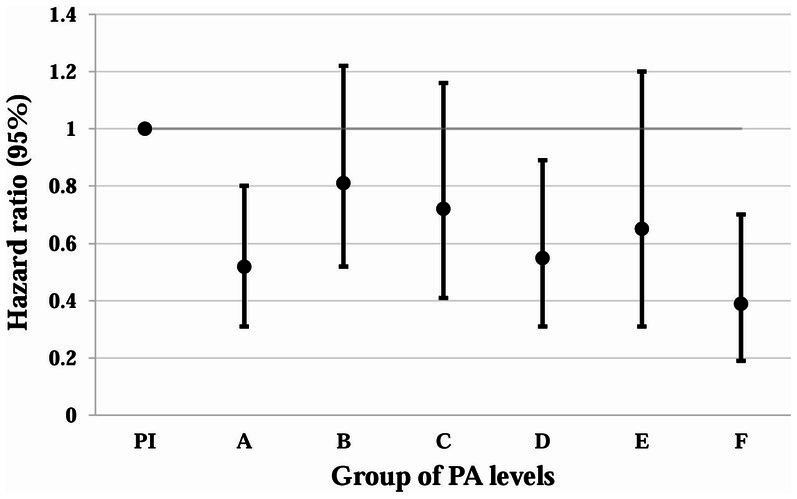
Association of different PAV with all-cause mortality in depressed patients. Whiskers represent 95% confidence intervals. PI: PAV < 600 (insufficient PA). A: 600 ≤ PAV ≤ 1,199. B: 1,200 ≤ PAV ≤ 1,799. C: 1,800 ≤ PAV ≤ 2,999. D: 3,000 ≤ PAV ≤ 5,999. E: 6,000 ≤ PAV ≤ 8,999. F: PAV ≥ 9,000. Unit: MET-min/week.

Table 3 and Figure 2 show the correlations between different PAV and the risk of all-cause mortality. After performing a more granular grouping of PA levels (Table 3; Figure 2), regardless of which group of PA levels is compared to <600 MET-min/week, the mortality rate decreases. In the fully adjusted Model 3, the risk of all-cause mortality was reduced by 48% with HR = 0.52 (95% CI: 0.31–0.80) for PA levels in the range of 600–1,199 MET-min/WEEK, after which the risk of mortality did not decrease more significantly with increasing PA levels. At PA levels 1,200–1,799, 1,800–2,999, 300–5,999, and 6,000–8,999, the risk of death decreased by 19, 28, 45, and 35%, respectively. The lower risk of death (HR = 0.39) that occurred for PAVs ≥9,000 MET-min/week may be due to bias from the small sample size.

**Table 3:** Association of different PAVs with all-cause mortality in depressed patients.

PAV (MET min/week)	Model 1	Model 2	Model 3
<600	Reference	Reference	Reference
600–1,199	0.48 (0.29–0.74)	0.48 (0.29–0.74)	0.52 (0.31–0.80)
1,200–1,799	0.81 (0.52–1.22)	0.77 (0.49–1.15)	0.81 (0.52–1.22)
1,800–2,999	0.57 (0.33–0.92)	0.64 (0.37–1.04)	0.72 (0.41–1.16)
3,000–5,999	0.45 (0.25–0.73)	0.50 (0.29–0.82)	0.55 (0.31–0.89)
6,000–8,999	0.56 (0.26–1.02)	0.61 (0.29–1.12)	0.65 (0.31–1.20)
≥9,000	0.32 (0.16–0.58)	0.34 (0.17–0.62)	0.39 (0.19–0.70)

Hazard ratios (95% confidence intervals [CIs]).

Model 1: Adjusted for gender + Age.

Model 2: Adjusted for Model 1 + Education, BMI, smoking status, and drinking status.

Model 3: Adjusted for Model 2 + Chronic illness.

### 3.3 Association of PA purpose with all-cause mortality in depressed patients

**Figure 3:**
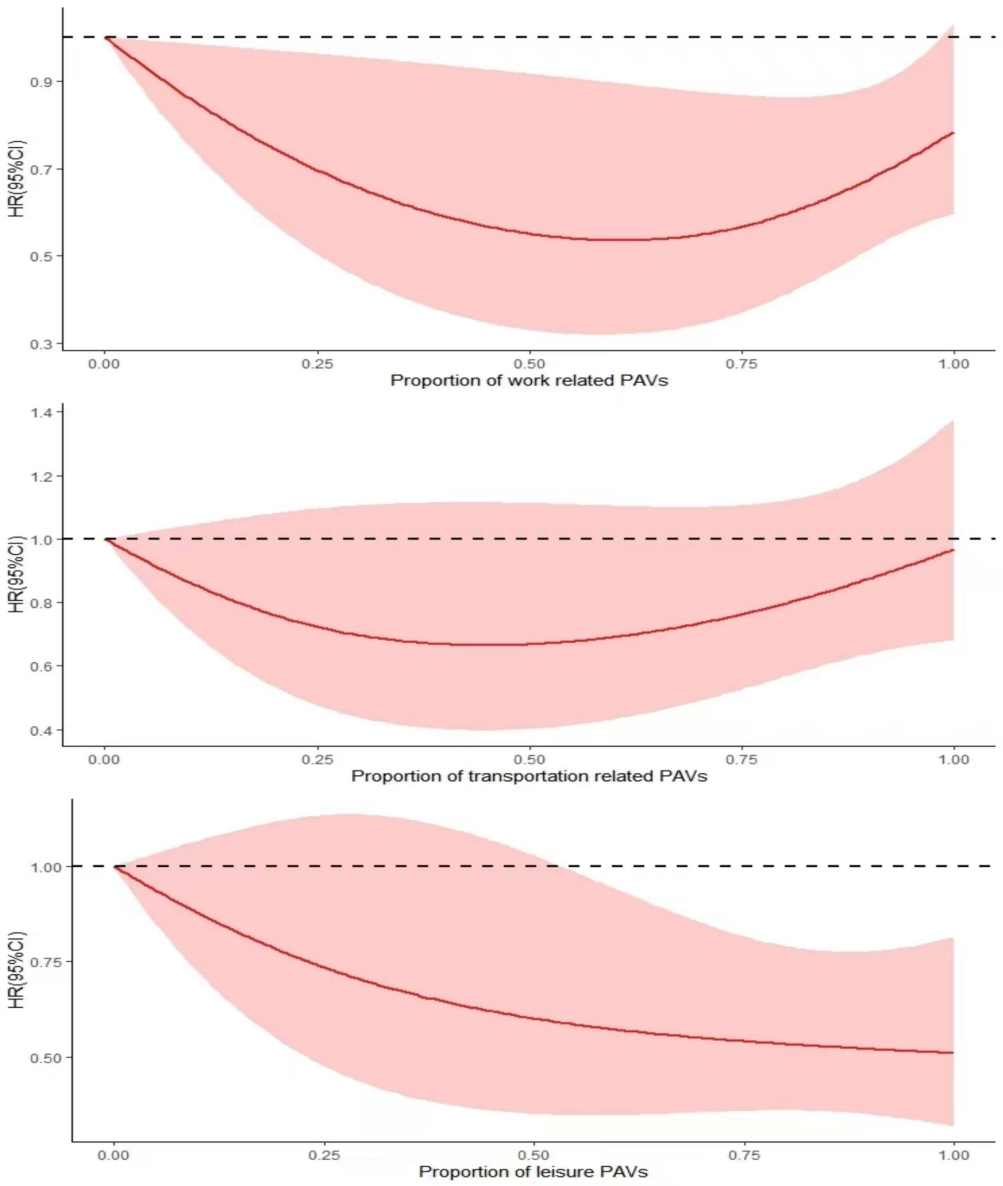
RCS plot of the association between different PA purposes and all-cause mortality in depressed patients.

In this study, PAs were classified according to different purposes (work or family, leisure time, and transport), with HRs (95% CIs) plotted on the vertical coordinate. The RCS plot, which is based on the percentage of different PA, revealed that all three types had the highest mortality rates among the subjects when no PA was performed (when the percentage of PA in the horizontal coordinate was 0). The work-type and transport-type PA had a gradually decreasing risk of death as the percentage of PA increased, reaching its lowest point of the risk of death when the percentage of PA in the horizontal coordinate reached approximately 0.5; this was followed by an upward trend, presenting a “U” curve. The leisure class PA proportion of the situation can be seen in the figure. The risk of death of depressed patients gradually decreases with increasing PA, and the higher the proportion is, the greater the effect of reducing the risk of death (Figure 3).

### 3.4 Subgroup analyses

Among the 2,841 depressed patients, 360 deaths occurred: 268 deaths (17.20%) among the 1,558 patients in the group lacking PA and 92 deaths (7.17%) among the 1,283 patients in the group undergoing sufficient PA, with an HR of 0.45 (95% CI: 0.36–0.57).

**Figure 4:**
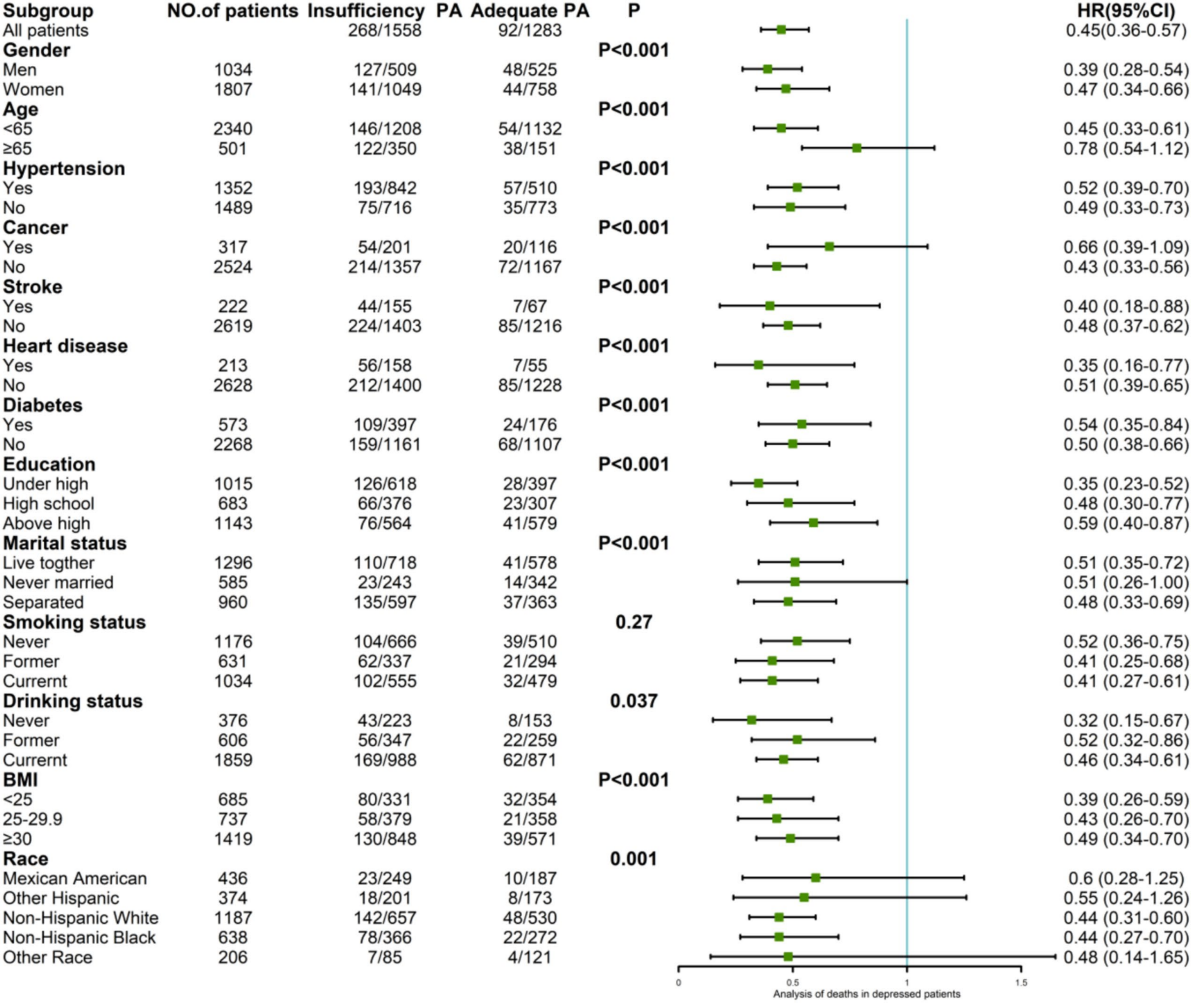
Subgroup analyses of the association between PA and all-cause mortality.

Subgroup analyses revealed variations in mortality risk reduction associated with sufficient PA. Among male participants, the HR was 0.39 (95% CI: 0.28–0.54), while among female participants, the HR was 0.47 (95% CI: 0.34–0.66). In the age-stratified analysis, individuals younger than 65 years had an HR of 0.45 (95% CI: 0.33–0.61), while no significant risk reduction was found in participants older than 65 years with HR = 0.78 (95% CI: 0.54–1.12). The participants with less than a high school education had the lowest HR of 0.35 (95% CI: 0.23–0.52). Those who had never consumed alcohol had an HR of 0.32 (95% CI: 0.15–0.67). Participants within the normal BMI range had an HR of 0.39 (95% CI: 0.26–0.59). Among individuals without hypertension, cancer, and diabetes, the HRs were 0.49 (95% CI: 0.33–0.73), 0.43 (95% CI: 0.33–0.73), and 0.50 (95% CI: 0.38–0.66), respectively. For those with stroke and heart disease, the HRs were 0.40 (95% CI: 0.18–0.88) and 0.35 (95% CI: 0.16–0.77), respectively (Figure 4).

## 4 Discussion

Among these screened subjects, an association analysis was conducted between physical activity and mortality to evaluate the impact of physical activity on mortality risk. The study revealed that, as long as sufficient physical activity is carried out, regardless of the amount of physical activity, the mortality risk of depressed patients can be reduced compared with that of the group lacking physical activity. Moreover, the proportion of leisure physical activities is negatively correlated with the risk of patient mortality. Even for depressed patients with stroke or heart disease, engaging in physical activity can reduce the risk of death.

This study found that sufficient physical activity significantly reduces mortality among individuals with depression. With an estimated 322 million people affected by depression worldwide, these findings highlight the vital role of PA in clinical and public health strategies to prevent deaths. Unhealthy behaviors such as being sedentary and physical inactivity are associated with a significantly increased risk of developing depression (38). The 2018 PA Guidelines (39) state that physical inactivity is one of the leading threats to human life and health, leading to increased depressive symptoms and an elevated risk of death (40). Physical activity is an intervention that can be effective in reducing the risk of death in patients with clinical depression (41). A growing number of articles and evidence suggest that a certain level of PA has a protective role in treating depression and reducing all-cause mortality. PA is the basis of primary prevention of depression and plays an important role in every age and stage of depression, especially with regard to depression in the older adult population (42). There is an inverse linear relationship between PA and the severity of depression (43). The majority of the studies, which are consistent with the results of this study, have shown that sufficient PA can reduce the risk of death in depressed patients compared with a lack of PA. Still, the discussion of whether different amounts of PA can improve depressive symptoms and reduce the risk of death is controversial. Studies have consistently shown that performing a small amount of PA has a better antidepressant effect when the frequency of exercise is appropriate (44). With respect to PA intervention as a complementary treatment for depressed patients, different levels of PA have different effects on depression (41, 45). For people who do not engage in physical activity, even at levels below those public health recommendations, the prevalence of depression decreases, as well as improvement in depressive symptoms. The benefits do not increase significantly with excessive levels of physical activity, and additional benefits are limited (25). Several experiments have shown that patients with depression can experience greater and better therapeutic effects when they undergo moderate-intensity physical activity experiments under the guidance of an exercise specialist (46); There may be an independent correlation between the level of PA and all-cause mortality in depressed patients (47). Increased exercise activity is inversely associated with the risk of illness and the incidence of depression (47). Trivedi and coworkers used two different doses, one being the upper limit of public health recommendations, and compared it with the lower limit of public health recommendations. The median adherence rate to low doses was significantly better than that to high doses. One study for major depression suggested that the high-dose group may have better cyclic relief for all men and women without a family history of mental illness. In addition, some Mendelian randomization studies validated a causal relationship between physical activity and depression (48, 49). However, exercise therapy dosage should be judged on an individual basis, and perhaps for some people, low-dose exercise may lead to better results instead (50). In addition to demonstrating the benefits of sufficient PA in reducing the risk of death in depressed patients, in the present study, different amounts of PA were found to reduce the risk of death, and a PAV of 600–1,199 MET-min/week may be sufficient to reduce the risk of death in depressed patients. In addition, the optimal amount of PA to reduce the risk of death occurring at ≥9,000 MET-min/week may be due to bias caused by the small sample size. In addition, it is possible that excessive amounts of PA may increase the risk of sports injuries and mental stress (51, 52). Only a few studies have explored the relationship between PA and mortality in individuals with depression. Murri et al. reviewed the mechanisms underlying the potential protective effects of PA on cardiovascular mortality in patients with depression (53). Luo et al. found that MVPA, but not LPA, reduced the risk of mortality associated with mood disorders, including depression (54). Expanding on this, the present study further investigates the association between PA volume and purpose with mortality in patients with depression. PA may reduce the mortality of patients with depression through physiological and psychological pathways (27). Biologically, it enhances neuroplasticity, reduces inflammation and oxidative stress, and regulates the HPA axis (27). Psychologically, it promotes self-esteem, strengthens social support, and enhances self-efficacy (27).

There is evidence that PA for different purposes influences the relationship between PA and depression (55). Leisure PA interventions appear to be best suited to improving levels of depression (56), reducing patients’ risk of all-cause mortality. This article follows the WHO classification of PA into three categories for different purposes (leisure time, work, and transport trips). It concludes that people with depression can try to engage in leisure-based PA. White et al. reported that excessive work-related PA was associated with increased levels of depression, whereas leisure-type PA led to decreased levels of depression (57). The majority of the work and transport categories are designed to achieve PA and serve as external motivators. To a certain extent, excessive PA produces positive benefits to reduce mortality risk. Still, excessive PA, instead, causes some physical and mental stress (51, 52), resulting in the same high mortality risk as a lack of PA. In contrast, activities performed during leisure time are more likely to be seen as self-directed, enjoyable tasks (57). According to self-determination theory, autonomous motivation related to PA is more likely to produce psychological satisfaction (autonomy, competence, and relatedness), which, in turn, supports mental health (58). The RCS graphs in the article indicate a tendency to increase the risk of death after a certain amount of physical activity has been exceeded, followed by a decrease in risk when engaging in more physical activity. However, if recreational physical activity is performed consistently, it may have a better positive effect.

In previous findings, BMI was found to be positively correlated with levels of depression, which increased as BMI increased (59). In particular, when depressed patients have physical inactivity and unhealthy eating habits leading to weight gain and a high BMI (60), the risk of death is significantly greater. Thus, in contrast, having a normal BMI and engaging in sufficient PA have positive effects on improving depressive symptoms and reducing the risk of death. Depression is a significant public health problem in older adults compared with middle-aged adults (61). There is a huge impact on mortality when it is comorbid with chronic diseases (62). Chronic diseases such as hypertension place a higher burden on depressed patients, which in turn leads to lifestyle changes such as PA deficiency, increasing the risk of eventual mortality (63). Another example is low cardiorespiratory fitness (CRF), which is an indicator of physical inactivity (23). In other meta-analyses, CRF was found to be negatively correlated with depression severity in depressed patients (64), and performing sufficient PA can increase CRF levels in patients, which is beneficial to the physical and mental health of depressed patients and prolongs survival time. The above extensive literature shows that depressed patients have an exponentially increased risk of death when accompanied by chronic diseases such as cardiovascular disease, cancer, and diabetes (65). In addition, sufficient PA can reduce depressive symptoms, increase CRF, increase muscle strength, and sustainably lower blood pressure (40), which indirectly reduces the associated risk of death to some extent.

This study underscores the crucial role of PA in reducing mortality among individuals with depression, highlighting its potential as a non-pharmacological intervention. The findings demonstrate that various intensities of PA contribute to lowering depression-related mortality. Clinical guidelines should incorporate PA recommendations tailored to individual needs, with a minimum of 600–1,199 MET-min per week to effectively reduce the risk of death. Mental health professionals and primary care providers should integrate PA counseling into routine care for individuals with depression, ensuring they have access to appropriate exercise programs. Public health policies should prioritize PA promotion through initiatives such as community-based exercise programs, workplace wellness strategies, and insurance coverage for PA-based treatments. These combined efforts can contribute to a more comprehensive and effective approach to addressing depression and its associated challenges.

### 4.1 Strengths and limitations

Strengths: First, this study involved a representative sample of the adult US population. Second, this was a longitudinal study in which the investigators followed up on the survival of the subjects. Finally, the US NHANES takes into account the national context and ensures the quality of the article’s data. Limitations: First, although data on variables such as PA in the NHANES database were collected by trained interviewers, participants’ responses to self-reported information may have been biased. Future research should consider using objective measures of PA, such as accelerometers. Second, changes in the course of the subjects’ depression during the follow-up years were not considered. Longitudinal studies tracking changes in depression severity over time could provide a clearer understanding of how fluctuations in depression influence the relationship between PA and mortality. Third, although we adjusted for multiple covariates in the analysis, the potential impact of unmeasured factors cannot be entirely excluded. Further research should investigate the impact of unmeasured confounders using advanced statistical methods or controlled trials to isolate the effect of PA on depression-related mortality more accurately.

## 5 Conclusion

This study showed that sufficient PA can significantly reduce all-cause mortality in patients with depression, with three main findings: (1) PAV between 600–1,199 MET-min/week are associated with a reduced risk of mortality; (2) Leisure-time PA demonstrates a dose-dependent inverse correlation with mortality, proving more beneficial than work- or transport-related activities; and (3) The protective effects of PA persist even in depressed patients with comorbidities such as stroke or heart disease. These findings reinforce the role of PA as a non-pharmacological intervention and highlight the need for clinical guidelines to prioritize leisure-time PA. However, this study has some limitations, including self-reported PA data, potential unmeasured confounders, and the absence of longitudinal depression tracking. Future research should use accelerometer-based PA measurements, monitor depression dynamics, and conduct randomized trials to optimize PA prescriptions. This study underscores the need to integrate tailored PA interventions into global depression management to reduce premature mortality in this vulnerable population.

## Data Availability

Publicly available datasets were analyzed in this study. This data can be found here: https://www.cdc.gov/nchs/nhanes/.
